# Identity-Preserved Human Posture Detection in Infrared Thermal Images: A Benchmark

**DOI:** 10.3390/s23010092

**Published:** 2022-12-22

**Authors:** Yongping Guo, Ying Chen, Jianzhi Deng, Shuiwang Li, Hui Zhou

**Affiliations:** Guangxi Key Laboratory of Embedded Technology and Intelligent Information Processing, College of Information Science and Engineering, Guilin University of Technology, Guilin 541006, China

**Keywords:** identity-preserved, human detection, infrared thermal images, human posture detection, benchmark, IPHPDT dataset

## Abstract

Human pose estimation has a variety of real-life applications, including human action recognition, AI-powered personal trainers, robotics, motion capture and augmented reality, gaming, and video surveillance. However, most current human pose estimation systems are based on RGB images, which do not seriously take into account personal privacy. Although identity-preserved algorithms are very desirable when human pose estimation is applied to scenarios where personal privacy does matter, developing human pose estimation algorithms based on identity-preserved modalities, such as thermal images concerned here, is very challenging due to the limited amount of training data currently available and the fact that infrared thermal images, unlike RGB images, lack rich texture cues which makes annotating training data itself impractical. In this paper, we formulate a new task with privacy protection that lies between human detection and human pose estimation by introducing a benchmark for IPHPDT (i.e., Identity-Preserved Human Posture Detection in Thermal images). This task has a threefold novel purpose: the first is to establish an identity-preserved task with thermal images; the second is to achieve more information other than the location of persons as provided by human detection for more advanced computer vision applications; the third is to avoid difficulties in collecting well-annotated data for human pose estimation in thermal images. The presented IPHPDT dataset contains four types of human postures, consisting of 75,000 images well-annotated with axis-aligned bounding boxes and postures of the persons. Based on this well-annotated IPHPDT dataset and three state-of-the-art algorithms, i.e., YOLOF (short for You Only Look One-level Feature), YOLOX (short for Exceeding YOLO Series in 2021) and TOOD (short for Task-aligned One-stage Object Detection), we establish three baseline detectors, called IPH-YOLOF, IPH-YOLOX, and IPH-TOOD. In the experiments, three baseline detectors are used to recognize four infrared human postures, and the mean average precision can reach 70.4%. The results show that the three baseline detectors can effectively perform accurate posture detection on the IPHPDT dataset. By releasing IPHPDT, we expect to encourage more future studies into human posture detection in infrared thermal images and draw more attention to this challenging task.

## 1. Introduction

Human pose or posture estimation has a variety of real-life applications, including human action recognition [1,2], AI-powered personal trainers [3,4], robotics [5,6], motion capture and augmented reality [7,8], gaming [9], video surveillance [10,11]. Traditionally, its purpose is to predict the positions of body joints from the input images, particularly in RGB modality. However, its ill-posedness aside, this task is challenging to generalize to those modalities short of texture information, such as infrared thermal images and depth images, since texture as an important visual cue plays a crucial role in identifying and localizing the joints. Human detection, as an upstream task to human pose or posture estimation, aims to locate all instances of human beings present in an image, usually involving both identifying the human beings and localizing the rectangular boundary surrounding each person. Nevertheless, despite vast applications such as safety, people flow, and surveillance [12,13,14], it provides only very limited information, i.e., no more than presence and localization, about the detected persons. In this paper, we motivate and formulate a task that lies between human pose estimation and human detection so that it generalizes well to broader application scenarios and provides more information as an upstream task for other applications.

Thanks to the great success of deep learning and available large-scale training data, human detection and localization technologies have advanced significantly in recent years. Currently, the majority of human detection is based on RGB images [15]; however, RGB images may show the private and social environment people are in and reveal their personal characteristics [16], severely threatening personal privacy and hindering its development in both scope and depth. Moreover, visible light images are more sensitive to light changes, weather changes, and other factors, which limits its application to some specific scenarios [17]. Therefore, non-RGB sensors, especially infrared thermal imaging sensors, are thus receiving increasing attention in human detection [18,19,20,21] and many other applications as well, such as station temperature measurement systems [22], medical diagnosis [23], and night patrol surveillance cameras [24]. Infrared thermography is a technology that combines optical and electronic technologies to distinguish from the environment by capturing infrared radiation from detected objects, and it can work in any environment and has a broader range of applications than visible light [25]. It is more challenging to perform human detection on infrared thermal images. Due to the relatively unique imaging mechanism and characteristics of IR thermal imaging, there are disadvantages such as blurred edge effect, fewer texture features, poor signal-to-noise ratio, and low resolution. Nevertheless, the advantages of infrared thermal images, such as being invariant to illuminating conditions, and robust to light variations and weather conditions, make infrared thermography an excellent alternative to RGB modality in many industrial, military, commercial, and medical applications. Importantly, providing fewer details makes it a good choice for applications where privacy protection matters, such as action recognition in hospitals [26], elderly healthcare applications [27,28] and privacy-preserving pedestrian detection [17]. Identity-preserved human detection has been attracting more and more attention recently [17,29,30]. Unfortunately, human detection can only provide the fundamental components of information that computer vision applications require, i.e., the locations of persons in a scene, knowing of which is insufficient for more complex computer vision tasks, however. For example, recognizing a person’s posture is crucial to extract high-level semantics for the task of scene understanding [31]. Estimating the pose of persons underpins various applications of human activity estimation, robotics, motion tracking, and augmented reality [5,7,8,32].

Human pose estimation is a way of identifying and classifying the human body’s joints in images. It generally uses the keypoint estimation method to select a set of most representative points in human pose [33,34], such as head, shoulders, elbows, wrists, hips, knees, ankles, and portray the human pose by connecting the lines. However, identifying these joints, especially manually, requires rich texture information. RGB modality meets this requirement very well, and plenty of algorithms have been proposed for human pose estimation based on RGB images [1,2,3,4,5,6]. Nevertheless, it is challenging to manually identify the joints of human bodies on modalities with less texture information, especially the thermal images concerned here. Developing and evaluating big deep-learning models is hardly possible without sufficient well-annotated data. Although RGB modality is usually combined with the ones with less texture information so that the annotations can be achieved by aligning the ones annotated on RGB images [17,30,35], the differences between RGB and some other modalities should not be neglected. For instance, the valid depth of field of an Intel RealSense D435 device is less than 60 m, much less than generic RGB cameras. Thermal sensors have a relatively low resolution; e.g., the images captured by the FLIR Lepton v3 sensor are of size only 213 × 120. These differences make the annotations by alignment questionable. Moreover, in privacy-sensitive scenarios, RGB images are hardly accessible. In short, human pose estimation in thermal images faces onerous challenges in collecting well-annotated data.

**Figure 1:**
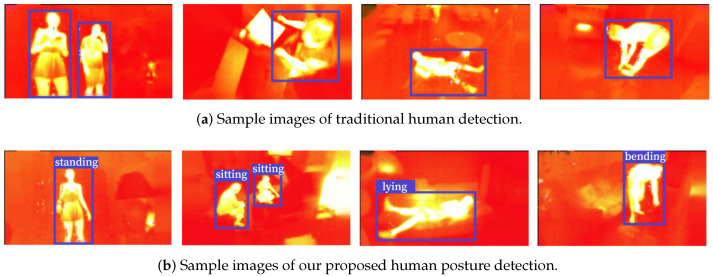
As illustratedin (**a**,**b**), respectively, the previous method of human detection concentrated on the identification and localization of humans; however, we also pay attention to additional information, i.e., the human posture. Note (**b**) marks the postures of the human (i.e., ‘standing’, ‘sitting’, ‘lying’, and ‘bending’ from left to right, respectively.) additionally.

Taken together, in order to solve the above three issues, i.e., to generalize the human detection task well to a broader range of application scenarios, to achieve more information other than the location of persons as provided by human detection for more advanced computer vision applications, and to avoid difficulties in collecting well-annotated data for human pose estimation in thermal images, we formulate a new task which is a compromise between human pose estimation and human detection. Specifically, in this paper, we focus on human posture recognition and localization in infrared thermal images, in which the human posture is divided into four common types, i.e., standing, sitting, lying, and bending. Our task is Identity-Preserved Human Posture Detection in Thermal images, for which we present a dataset to facilitate future research called the IPHPDT dataset. An illustration of the distinction between traditional human detection and our human posture detection in infrared thermal images is shown in Figure 1, where Figure 1a is from IPHD dataset [17] and Figure 1b is from IPHPDT dataset. The IPHPDT dataset contains four types of human posture of bounding-box annotations collected from 75,000 infrared thermal images. On the basis of this well-annotated dataset, we have established three baselines based on three state-of-the-art algorithms, i.e., IPH-YOLOF, IPH-YOLOX, and IPH-TOOD. As the posture of persons being provided, this task has great significance for the extension of application scenarios, for example, AI-powered personal trainers [3,4], gaming [9], video surveillance [10,11], robotics [5,6], and so on, particularly for applications with privacy protection by the thermal modality. We believe this task also has far-reaching implications in computer vision perception, analysis, and interpretation and may lead to further exploration of new detection tasks beyond identification and localization.

**Figure 2:**
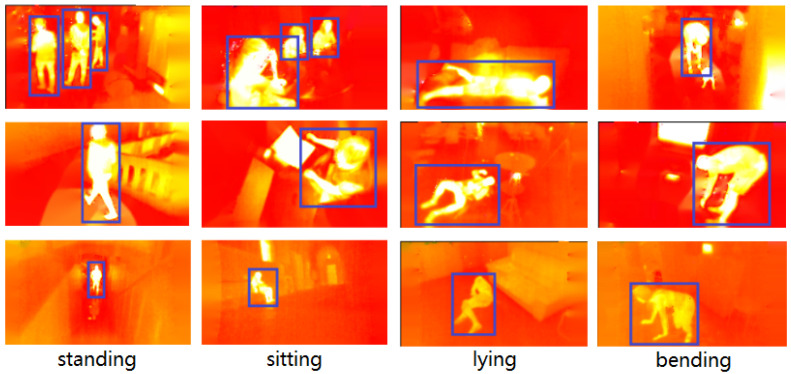
Samples images of persons with four human postures (i.e., ‘standing’, ‘sitting’, ‘lying’, and ‘bending’ from left to right) in the proposed IPHPDT dataset. The objects are identified by blue bounding boxes.

In this work, we formulate a new task with privacy protection that lies between human detection and human pose estimation by introducing the IPHPDT benchmark for identity-preserved human posture detection in thermal images. The IPHPDT dataset consists only of human objects, and it contains 75,000 images with axis-aligned bounding boxes and postures of the persons. Figure 2 shows some sample images in the IPHPDT dataset. Additionally, we developed three baseline detectors, i.e., IPH-YOLOF, IPH-YOLOX, and IPH-TOOD, based on three state-of-the-art detectors, i.e., YOLOF, YOLOX, and TOOD, to make sense of the performance of the task and to offer comparisons for IPHPDT study in the future.

Our contributions are summarized as follows,

- We formulate a novel task of identity-preserved human posture detection in thermal images, which underpins various applications where privacy matters and which may also draw attention to more informative object detection other than identification and localization.
- We present the IPHPDT dataset, which is the first benchmark dedicated to identity-preserved human posture detection in thermal images.
- We develop three baseline detectors based on three state-of-the-art detectors, i.e., YOLOF, YOLOX, and TOOD, to facilitate and encourage further research on IPHPDT.

## 2. Related Work

### 2.1. Traditional Methods of Human Detection in Infrared Thermal Images

In traditional human detection methods of infrared thermal images, many researchers were keen on using human grayscale values for human detection and localization. For instance, Comaniciu et al. [36] proposed an infrared human target tracking algorithm based on the Mean Shift algorithm, which identifies the human target by the unique grayscale value characteristics of the human and simplifies the target tracking problem by using the solving process of the optimal solution, and the Bhattacharyya Coefficient is also introduced as a judgment value, which measures the approximation of the current model to the candidate model. Nanda et al. [37] proposed a human detection model based on the grayscale value of infrared thermal images by using the values related to the human target, such as the grayscale mean to calculate the grayscale value threshold of the human, distinguishing the region of interest by dividing the region, and finally constructing a grayscale value probability model for human detection. Later, combining thermal features with other human features for human detection gradually became mainstream. Fernández-Caballero et al. [38] proposed a thermal-infrared pedestrian ROI extraction algorithm that fuses human thermal features and motion information. Zheng et al. [39] proposed a mutual guidance method based on saliency propagation for infrared pedestrian images, simultaneously using human thermal and appearance features. Zhang et al. [40] proposed an association saliency segmentation method for infrared targets, and this association saliency is generated by region saliency and margin contrast. A visual attention model using the saliency detection method to improve the accuracy of target segmentation and detection in infrared thermal images. Although these traditional methods once provided an impetus to the development of human detection on thermal images, their performances hardly match that of deep learning-based ones that were proposed recently.

### 2.2. Deep Learning Methods for Human Detection Based on Infrared Thermal Images

Due to the high efficiency of deep learning in deep mining features of images, many researchers are keen to use deep learning networks for infrared thermal imaging human detection. For instance, Biswas et al. [41] proposed to apply linear support vector machines to human detection in thermal infrared scenes. They established linear support tensor machines with LSK channels and used Local Steering Kernel (LSK) as low-level descriptors to detect human bodies in far-infrared thermal images for fast and effective human detection and localization. Tan et al. [42] proposed to use a Multi-scale Monogenic Signal representation of feature descriptors and a “deep brief network” for thermal infrared human recognition, which can improve recognition accuracy and robustness to landscape changes. Based on deep learning, many researchers are keen to apply improved CNN to thermal infrared human recognition to improve the accuracy of human posture recognition in complex scenes. For instance, Akula et al. [43] proposed a deep learning approach to recognize human actions in infrared human thermal images and designed a two-layer convolutional neural network architecture with supervision that is capable of recognizing six human actions. Wu et al. [44] combined temporal and spatial convolution and proposed an algorithm based on a spatio-temporal dual-stream convolutional neural network, which is able to process longer videos and fully consider video information and perfusion information to improve the accuracy of human action recognition in infrared video. In the CNN series, the emergence of a one-stage algorithm represented by YOLO makes object detection faster, and it can predict the whole image, so its application to infrared images for human detection is gradually becoming a research hotspot. For instance, Ma et al. [45] proposed an improved YOLO v3 algorithm and applied it to infrared image pedestrian detection. They used k-means++ [46] clustering algorithm to re-cluster anchor boxes of the pedestrian dataset, used GIoU instead of mean squared difference as the border loss function, and removed the convolution layer in front of the multi-scale detection end of the network structure. Shi et al. [47] proposed an improved YOLO v4 infrared image pedestrian detection algorithm. They used deformation convolution to improve the effectiveness of target feature extraction, added coordinates to the attention mechanism module to enhance the coordinate information, and increased a “Guided Anchoring” mechanism to the detection layer to improve the accuracy of network localization.

Overall, in the field of object detection using a single neural network with object detection as a regression task with spatially separated bounding boxes and associated class probabilities, YOLO dominates and is the most widely used method with numerous variants as a result of its quickness, accuracy, and learning capabilities. In view of this, we adopt the two most recent YOLO variants and one TOOD variant in this paper to build our baselines for identity-preserved human posture detection in thermal images.

### 2.3. Human Pose Estimation

Generally, Human Pose Estimation can be subdivided into 2D/3D Pose Estimation. The main task of 2D human pose estimation is to locate and detect the human body keypoint, thus obtaining the human body skeleton; however, the main task of 3D human pose estimation is to predict the 3D coordinates and angles of the human body joints. Indeed, these two tasks are closely related. Every 3D pose can be projected to a 2D pose, and a 3D pose can also be inferred using 2D pose estimation [48]. Most current Human Pose Estimation algorithms are focused on predicting the coordinates of human keypoint, i.e., keypoint localization, which portrays the human pose by determining the spatial location relationship between keypoints through a priori knowledge. For example, Zhang et al. [49] designed a new network architecture to achieve high performance in Human Keypoint Detection. They integrated contextual information to infer the human body and hard keypoints by cascading contextual mixers (CCM) and developed two strategies to maximize the representation capability of CCM, besides proposing some sub-pixel refinement techniques to improve localization accuracy. However, identifying human body keypoints, especially manually identifying them, requires rich texture information. Therefore most researchers are currently performing Human Pose Estimation based on RGB images [1,2,3,4,5,6] since RGB images are more favorable in this regard. Unfortunately, RGB images are prone to infringe upon personal privacy, hindering their application in fields where privacy does matter. So, there is a pressing need to develop pose estimation algorithms based on modalities that can preserve personal identity other than RGB, which motivates us to consider human pose estimation based on thermal images that have proven to be well identity-preserved [17]. Nevertheless, manually identifying human body joints on thermal images is very difficult. Developing and evaluating big deep-learning models is hardly possible without sufficient well-annotated data. Although RGB modality is usually combined with thermal images so that the annotations can be achieved by aligning the ones annotated on RGB images [17,35], the differences between them can not be neglected. In view of these, in this paper, we propose a novel and privacy-preserving oriented task that lies between human pose estimation and human detection, namely identity-preserved human posture detection in thermal images. The comparison of our method with previous approaches in terms of the used dataset, learning method, supervision method, use of YOLO, attention mechanism, FPN, and posture prediction head (or not) is summarized in Table 1.

## 3. Benchmark for Detecting Posture of Human

Our goal is to develop a dataset for Identity-preserved Human Posture Detection in Thermal images (IPHPDT). Since there exist datasets for human detection in thermal images, we do not intend to construct our dataset from scratch, which is time-consuming, expensive, and effort-taking. Instead, we build our dataset based on the identity-preserving human detection (IPHD) dataset [17], which is the most extensive thermal human body dataset to date.

### 3.1. IPHPDT Collection

The proposed IPHPDT dataset is obtained by selecting and then re-annotating thermal images of the multimodal image dataset IPHD [17]. The thermal images in the dataset were captured by a FLIR Lepton v3 sensor, with each pixel in thermal images representing the absolute temperature measured (in degrees Kelvin (K) multiplied by 100). The scenarios in this dataset include a mixture of public, private space, and wild pedestrian scenes from near and far, in which people behave differently, covering a wider range of postures, clothing, lighting, ambient temperature, cluttered backgrounds, and obscured spaces. Each image in IPHD has been annotated with axis-aligned bounding boxes. In developing IPHPDT based on IPHD, we cover the four most common human postures in daily lives, i.e., ‘standing’, ‘sitting’, ‘lying’, and ‘bending’. See Figure 2 for sample images of our dataset.

The IPHD train set has 84,818 images, from which we selected images with clear human target and human posture that is relatively easy to be distinguished. The IPHD validation set and test set have 12,974 and 15,115 images, respectively; we first combined them into one test set and then removed the images without a human target or the images with the human posture that cannot be distinguished relatively easily. Next, we counted the number of the four postures in the train set and test set initially divided above and found that the distribution of postures was unbalanced. Thus, we randomly extracted some posture data from each train set and test set and performed the swap operation to make the ratio of the four postures in the train set, and test set balanced to form the final IPHPDT dataset proposed by us. Finally, the IPHPDT train set has 62,010 images, and the test set has 13,267 images. See Table 2 for comparing the number of images between the IPHD dataset and our proposed IPHPDT dataset.

### 3.2. Annotation

The IPHPDT dataset annotation process is described in this section. According to the proposed task of detecting human posture, image annotations require the following attributes.

- **category:** person.
- **bounding box:** a bounding box with axis-alignment around the visible human in the image.
- **human posture:** one of standing, sitting, lying, and bending.

According to the annotation guidelines [55], there are three steps to our annotating process, i.e., manual annotation, visual inspection, and box refinement. Since the IPHD dataset has provided bounding box annotations, we only need to annotate the posture of each person in the images in the first stage, except that we may adjust the original bounding boxes we thought were not accurate enough. In the second stage, we send the data to a dedicated validation team for visual inspection and send the annotations that most people disagree with to the initial annotator for carrying out refinement operation in the third stage. After the above three-stage strategy, it is possible to ensure high-quality annotation of targets in the IPHPDT. Some examples of box annotations in the IPHPDT are shown in Figure 2.

### 3.3. Image Processing

**Figure 3:**
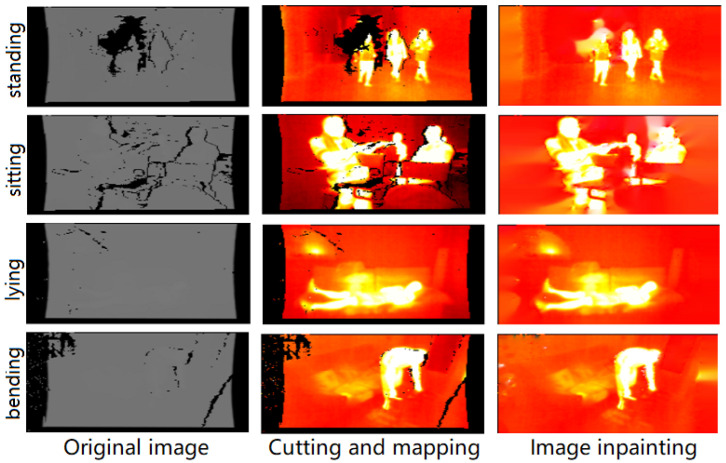
An illustration of the visualization results before and after image processing. The **left** column shows the original input images, the middle column shows the images after cutting and mapping, and the **right** column shows the images after image inpainting.

Since the thermal images in the IPHD dataset are registered to the corresponding depth images, the thermal frames may contain zero-valued pixels incorrectly derived from the depth errors, generating many unreasonable temperature readings, which would result in an increase in the range of thermal pixels and compressing more reasonable thermal readings. See Figure 3 for an illustration. As can be seen, the original thermal images are visually much less informative than RGB images. Directly using original thermal images to train a detector may incur severe domain-shift problems, as the backbone of the detector is usually pretrained by large-scale RGB images. Therefore, image enhancement is performed here to relieve the impact of domain shift. Our image enhancement consists of two steps. First, we perform soft classification of the pixel (temperature) clusters using a Gaussian Mixture Model(GMM) [56] to find the optimal temperature range for each image, cutting out the unreasonable thermal readings and then mapping the temperatures to the RGB color space. Second, we use the image inpainting method proposed in [57] to perform the image restoration. We use enhanced images as the input data for training in this paper. Some resulting examples of our image processing of the IPHPDT are shown in Figure 3. Note that ’Cutting and mapping’ indicates the results after the first step while ’Image inpainting’ indicates the results after the second step.

### 3.4. Dataset Statistics

**Figure 4:**
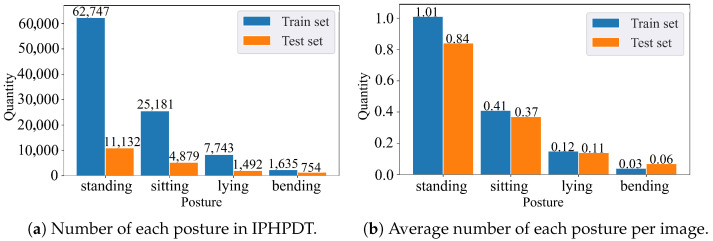
(**a**) shows the number of each human posture in the train set and test set on the IPHPDT dataset, and (**b**) shows the average number of each human posture per image in the train set and test set on the IPHPDT dataset.

In order to promote training and evaluation, the IPHPDT dataset is split into two primary subsets, i.e., train set and test set, with a ratio of 8/2. The statistics of the IPHPDT dataset are summarized in Figure 4. Figure 4a displays the number of each human posture in the train set and test set on the IPHPDT dataset, Figure 4b displays the average number of each human posture per image in the train set and test set on the IPHPDT dataset. As can be seen, the distribution of the four human postures in IPHPDT varies widely due to the unevenness of the source IPHD data, suggesting that standing and sitting postures have multiple targets in an image in most cases while lying and bending postures have basically a single target or absent in one image. Although it is worrying about encountering an imbalance problem of samples here, the imbalance of posture reflects the actual distribution of human posture in daily life, and our experimental results dispel this anxiety.

## 4. Baseline Detectors for Detecting Human Posture in Thermal Images

To facilitate the development of human posture detection in thermal images, we propose three baseline detectors based on three state-of-the-art object detection algorithms, i.e., variants of YOLOF [58], YOLOX [59], and TOOD [60]. We add an additional posture prediction head to each original model to predict the per person’s posture, resulting in three new detectors, which are dubbed IPH-YOLOF, IPH-YOLOX and IPH-TOOD, respectively. The details of the three baseline detectors are described in detail in the following.

### 4.1. IPH-YOLOF

**Figure 5:**
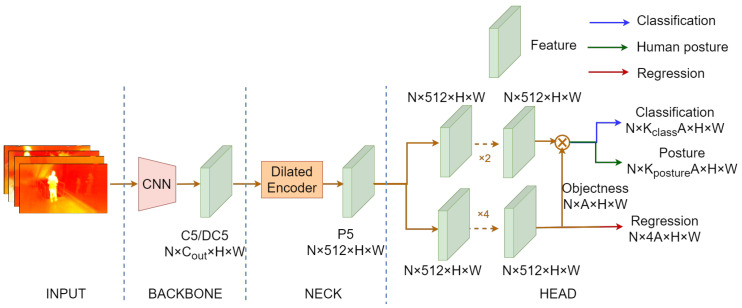
The generalstructure of the IPH-YOLOF detector we proposed. The network structure is a carryover from YOLOF [58] with the difference of an extra head that is utilized to predict human posture.

Our proposed network structure of IPH-YOLOF is shown in Figure 5. IPH-YOLOF uses the classical Resnet50 [61] as the backbone network, which is pre-trained on ImageNet [62]. The C5/DC5 feature map output by the backbone network has 2048 channels and a downsampling multiplicity of 32/16. These features are sent to a dilated encoder in the neck sub-network, responsible for the encoding process. The final decoding part contains two concurrent task-related heads for classification and regression, to which we add an extra prediction head for human posture prediction. The following is the definition of the total loss of training IPH-YOLOF,

(1) $$L_{total}=L_{cls}+L_{reg}+\lambda L_{posture},$$

where $L_{cls}$, $L_{reg}$, and $L_{posture}$ indicate the losses of classification, regression, and human posture prediction, and λ indicates the weight coefficient of loss for the human posture prediction head. The following are definitions of these losses, referenced from [63],

(2) $$L_{cls}=\frac{1}{N_{pos}}\sum_{n=0}^{N_{pos}}FL\left(y_{cls}^{n},p_{cls}^{n}⊗p_{obj}^{n}\right),\;L_{reg}=\frac{1}{N_{pos}}\sum_{n=0}^{N_{pos}}smooth_{L_{1}}\left(b_{t}^{n}-b_{p}^{n}\right),$$

(3) $$L_{posture}=\frac{1}{N_{pos}}\sum_{n=0}^{N_{pos}}FL\left(y_{posture}^{n},p_{posture}^{n}⊗p_{obj}^{n}\right),$$

where $y_{cls}^{n}$ and $y_{posture}^{n}$ indicate ground truth for the classification and human posture, and $p_{cls}^{n}$, $p_{posture}^{n}$, and $p_{obj}^{n}$ indicate the predictions for the classification, human posture, and boxes (i.e., is there any person in the box). FL(·) and $smooth_{L_{1}}$ indicate the focal loss and the $smooth_{L_{1}}$ loss functions, respectively. The focal loss function is mainly used to solve the problem of imbalance between difficult and easy samples by increasing the weight of the small number of target categories and misclassified samples. The $smooth_{L_{1}}$ loss function is insensitive to outliers (meaning points far from the center) and makes the training less prone to the gradient explosion by controlling the magnitude of the gradient. $N_{pos}$ indicates the amount of positive anchor, ${⨂}_{p}$ indicates the scalar product, and $b_{t}^{n}$ and $b_{p}^{n}$ indicate the ground truth bounding box and the prediction bounding box, respectively.

### 4.2. IPH-YOLOX

**Figure 6:**
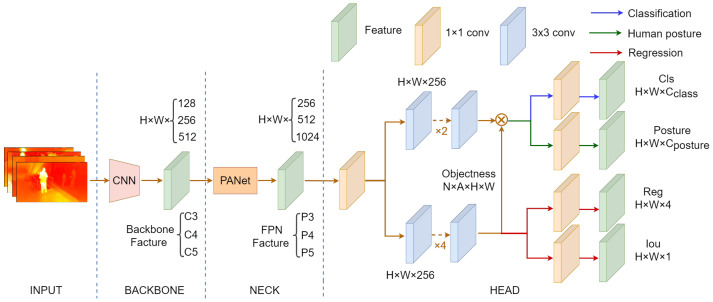
The generalstructure of the IPH-YOLOX detector we proposed. The network structure is a carryover from YOLOX [59] with the difference of an extra head that is utilized to predict human posture.

Our proposed network structure of IPH-YOLOX is shown in Figure 6. IPH-YOLOX uses the classical CSPDarkNet and the Spatial Pyramid Pooling(SPP) [64] layer as the backbone network. The C3, C4, and C5 features output by the backbone network have 128, 256, and 512 channels with downsampling multipliers of 8, 16, and 32, respectively. These features are sent to an enhanced feature extraction network PANet [65] in the neck sub-network. Those deep features are first fused with shallow features by a bottom-up path and then with deep features by a top-down path. The final decoupling part contains two concurrent task-related heads for classification and regression, to which we add an extra prediction head for predicting human posture. The following is the definition of the total loss of training IPH-YOLOX,

(4) $$L_{total}=L_{cls}+L_{reg}+L_{obj}+\lambda L_{posture},$$

where $L_{cls}$, $L_{reg}$, $L_{obj}$, and $L_{posture}$ indicate the losses of classification, regression, the confidence of boxes, and prediction of human posture, and λ indicates the weight coefficient of loss for the human posture prediction head. The following are definitions of these losses, referenced from [63],

(5) $$L_{obj}=\frac{-1}{N_{pos}}\sum_{n=1}^{N_{pos}}y_{obj}^{n}\ln\left(\sigma \left(p_{obj}^{n}\right)\right),\;L_{reg}=\frac{1}{N_{pos}}\sum_{n=1}^{N_{pos}}\left(1-IOU\left(b_{t}^{n},b_{p}^{n}\right)\right),$$

(6) $$L_{cls}=\frac{-1}{N_{pos}}\sum_{n=1}^{N_{pos}}y_{cls}^{n}\ln\left(\sigma \left(p_{cls}^{n}\right)\right),\;L_{posture}=\frac{-1}{N_{pos}}\sum_{n=1}^{N_{pos}}y_{posture}^{n}\ln\left(\sigma \left(p_{posture}^{n}\right)\right),$$

where $y_{cls}^{n}$, $y_{posture}^{n}$, and $y_{obj}^{n}$ indicate ground truth for the classification, human posture, and boxes, and $p_{cls}^{n}$, $p_{posture}^{n}$, and $p_{obj}^{n}$ indicate the predictions of classification, human posture, and boxes. σ and IOU(·) indicate the softmax activation and the IOU loss functions. IOU can be used to determine positive and negative samples and evaluate the distance between the prediction bbox and ground truth bbox, which has the property of scale invariance. $N_{pos}$ indicates the amount of positive anchor, and $b_{t}^{n}$ and $b_{p}^{n}$ indicate the ground truth bounding box and the prediction bounding box, respectively.

### 4.3. IPH-TOOD

**Figure 7:**
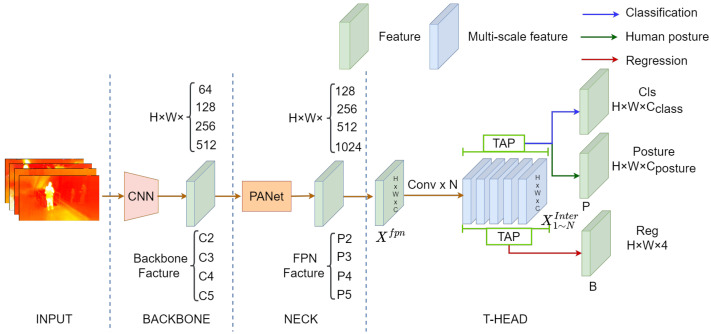
The generalstructure of the IPH-TOOD detector we proposed. The network structure is a carryover from TOOD [60] with the difference of an extra head that is utilized to predict human posture.

Our proposed network structure of IPH-TOOD is shown in Figure 7. The backbone of IPH-TOOD is also the Resnet50. The C2, C3, C4, and C5 features output from the backbone network have 256, 512, 1024, and 2048 channels with downsampling multipliers of 4, 8, 16, and 32, respectively. These features are sent to an FPN network in the neck sub-network, which is used to fuse multi-scale features output from the backbone network. There is a Task-aligned predictor (TAP) in the neck to adjust the features for task-specific heads, which in the original TOOD consists of the classification head and the regression head. In IPH-TOOD, we add an extra prediction head for human posture prediction. The following is the definition of the total loss of training IPH-TOOD,

(7) $$L_{total}=L_{cls}+L_{reg}+\lambda L_{pose},$$

where $L_{cls}$, $L_{reg}$, and $L_{pose}$ indicate the losses of classification, regression, and human posture prediction, respectively, and λ indicates the weight coefficient of loss for the human posture prediction head. The following are definitions of these losses, referenced from [60],

(8) $$L_{reg}=\sum_{i=1}^{N_{pos}}{\hat{t}}_{i}L_{GIOU}\left(b_{i},{\bar{b}}_{i}\right),$$

(9) $$L_{cls}=\sum_{i=1}^{N_{pos}}{|{\hat{t}}_{cls,i}-s_{cls,i}|}_{}^{\gamma }BCE\left(s_{cls,i},{\hat{t}}_{cls,i}\right)+\sum_{j=1}^{N_{neg}}{s_{cls,j}}_{}^{\gamma }BCE\left(s_{cls,j},0\right),$$

(10) $$L_{pose}=\sum_{i=1}^{N_{pos}}{|{\hat{t}}_{pose,i}-s_{pose,i}|}_{}^{\gamma }BCE\left(s_{pose,i},{\hat{t}}_{pose,i}\right)+\sum_{j=1}^{N_{neg}}{s_{pose,j}}_{}^{\gamma }BCE\left(s_{pose,j},0\right),$$

where $s_{cls}$ and $s_{pose}$ are the classification and human posture scores, respectively. $\hat{t}$ is the normalized *t*, and *t* denotes the anchor-level alignment and

$$t_{cls}=s_{cls}^{\alpha }+u_{}^{\beta }$$

and

$$t_{pose}=s_{pose}^{\alpha }+u_{}^{\beta },$$

where *u* denotes the IoU value, and α and β are the weights, respectively. BCE and $L_{GIOU}$ indicate the Binary Cross Entropy loss function and the Generalized Intersection over the Union loss function. BCE uses the sigmoid activation function, which can account for both positive and negative sample losses. $L_{GIOU}$ focuses on both overlapping and non-overlapping regions, which can solve the problem that the gap between non-overlapping frames cannot be evaluated. $N_{pos}$ and $N_{neg}$ indicate the amount of positive anchor and negative anchor, respectively. *i* indicates the *i*-th anchor from the $N_{pos}$ positive anchors corresponding to one instance, and *j* indicates the *j*-th anchor from the $N_{neg}$ negative anchors corresponding to one instance. γ indicates the focusing parameter, and $b_{i}$ and ${\bar{b}}_{i}$ indicate predicted bounding boxes and the corresponding ground truth bounding boxes, respectively.

## 5. Evaluation

### 5.1. Evaluation Metrics

In the experiment, we use AP (i.e., Average Precision) and mAP (i.e., mean Average Precision) to measure the performance of the three baseline detectors, besides using IOU (i.e., Intersection of Union) to measure the degree of error between ground truth bbox and predicted bbox. TP (True Positive), TN (True Negative), FP (False Positive), and FN (False Negative) are the number of pixels in the detection that match the ground truth (for TN/TP) or do not (FP/FN). For a detailed description, please refer to [55]. The following are the definitions of these evaluation indicators,

(11) $$AP=\frac{1}{11}\sum_{r\in \{0,0.1,\ldots ,1\}}\max_{\tilde{r}:\tilde{r}\geq r}p\left(\tilde{r}\right),\;mAP=\frac{\sum_{i=1}^{k}{AP}_{i}}{k},$$

(12) $$Precision=\frac{TP}{TP+FP},\;Recall=\frac{TP}{TP+FN},$$

In the general convention of computer vision, 0.5 is often set as the threshold to determine whether the predicted bounding box is correct, while we follow the COCO evaluation metric [66] to evaluate it. In COCO evaluation, the IoU threshold is divided into three metrics, which are 0.5, 0.75, and 0.5 to 0.95, respectively. When IoU = 0.5 and IoU = 0.75, the corresponding AP is expressed as AP@0.5 and AP@0.75; when the IOU is between 0.5 and 0.95, the step size is 0.05, and the corresponding AP is expressed as AP@[.50:.05:.95]. To assess the detector’s performance in detecting human posture, we used the COCO mAP metric. In general object detection, precision only predicts the accuracy of the target category. However, our task concerns the measurement of different postures of human, which requires the combination of two tasks, i.e., the traditional task of person detection and the new task of prediction of different human postures, which means that the precision metric for our task has to take into account predicting both the category and the posture at the same time. As a matter of convenience, the precision metrics for the prediction of the human category, human posture, and the composite of the two are represented by the $AP_{c}$, $AP_{p}$ and $AP_{cp}$, respectively, besides by adding a prefix ’m’ we indicate the mean AP, i.e., mAP.

### 5.2. Evaluation Results

**Overall performance.** We performed an extensive evaluation of the IPHPDT dataset using the three baseline detectors we proposed, i.e., IPH-YOLOF, IPH-YOLOX, and IPH-TOOD. As shown in Table 3, the precision metrics $AP_{c}$, $AP_{p}$, and $AP_{cp}$ listed in Section 5.1 are used to report the evaluation results. It is clear that IPH-TOOD is essentially the best detector, except that all its $AP_{c}$s are slightly lower than those of IPH-YOLOX and its $AP_{p}@$0.5 and $AP_{cp}@$0.5 are slightly lower than that of IPH-YOLOF. Compared with IPH-YOLOX, IPH-YOLOF is overall superior, except for its $AP_{c}$s being slightly lower than those of IPH-YOLOX. From Table 3, we can also observe that the average precisions of predicting human category are greater than the prediction of human posture for all three detectors. Specifically, the differences between $mAP_{c}$ and $mAP_{p}$ are all larger than 7.5%, with the OS-YOLOX detector seeing the largest difference of 11.2%, which indicates that it is more difficult and challenging to detect human posture than detect human itself in thermal images. That may be explained by the fact that distinguishing a person from the background is easier than distinguishing which posture a person is holding since the latter confronts much smaller inter-class differences. Although facing more challenges, we believe the proposed task has a broad application prospect, and our work will inspire more researchers to work on human posture detection in thermal images.

**Performance on per posture.** We evaluate the performance of the three proposed baseline detectors on each posture to further analyze and understand the performance of human posture detection in thermal images. Table 4 displays the $mAP_{cp}$ of the three detectors. It can be seen that the three detectors perform best in standing, followed by lying, with both $mAP_{cp}$ above 70%, but the $mAP_{cp}$ of sitting and bending are all below 70%. This can be attributed to the fact that: (1) standing is the posture of the largest amount of training data; (2) standing and lying facing less intra-class variations than sitting and bending do due to potentially more occlusion, and larger posture variations, for the latter. More specifically, in the IPHPDT dataset, even if the amount of data for sitting is only second to standing, it is subject to more occlusion and sitting posture variations, making detecting the sitting posture very challenging. Although with the least training data, the lying posture is basically a comparatively clear single-object present in each image, making detecting it relatively simpler. As to the bending posture, in addition to possible occlusion, when the bending angle is small, the detectors are prone to confuse it with the standing posture, thus leading to poorer test results. In our future work, we will account for these factors to develop better detectors for detecting human posture in thermal images.

**Figure 8:**
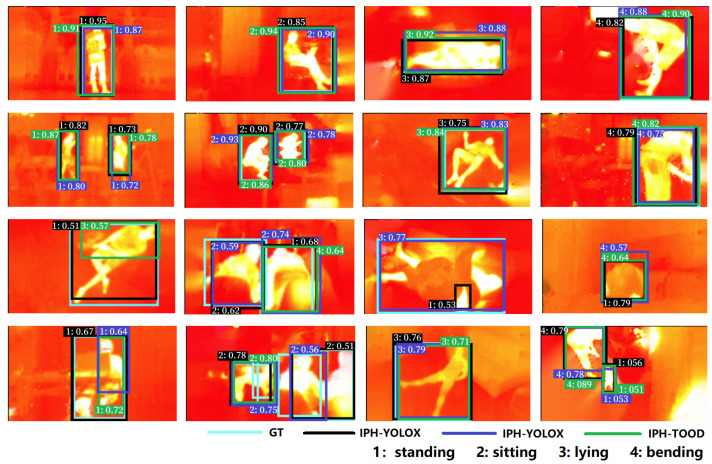
Qualitative evaluation of 16 samples in IPHPDT. The first two rows show examples of our proposed three detectors correctly predicting human postures. The last two rows show examples that our proposed three detectors fail to correctly predict human postures. Note that the number before the colon indicates the predicted posture, i.e., 1, 2, 3, and 4 represent standing, sitting, lying, and bending, respectively. GT stands for ground truth.

**Qualitative evaluation.** Qualitative detection results of 16 samples by the three proposed detectors are shown in Figure 8. The first two rows demonstrate eight examples on which the three baseline detectors perform very well, while the last two rows show eight examples that the detectors fail to predict human posture correctly. Where the black, blue, and green bounding boxes indicate the detection results of TPH-YOLOF, TPH-YOLOX, and TPH-TOOD, respectively. For the number on the boxes, e.g., 1: 0.91, the integer before the colon indicates the predicted human posture category (i.e., 1, 2, 3, and 4 represent standing, sitting, lying, and bending), and the decimal number after the colon indicates the predicted score. The three baseline detectors perform well on the first two rows because no occlusion, less background cluster, and of standard target size characterize the images in the first two rows. However, in the images in the last two rows, human postures are predicted incorrectly due to occlusion, background cluster, low-contrast of infrared images, and ambiguity. We take the last row as an example to analyze the possible causes of detection errors. From left to right, the first and the third samples are confused by the detectors due to occlusion, lack of clear texture features, and ambiguity, leading to errors in the detection of the posture type; the second is an example of false and missed detection, due to cluster, occlusion, and small object, resulting in missed detection of sitting postures; the fourth sample incorrectly detects the dog as a human, which may be due to the fact that in the infrared thermal images, the human thermal feature is mainly used as one of the most effective features to characterize the human body, thus when the object temperature is too high or even beyond the human body temperature, it may be wrongly detected as human. These results suggest that under challenging conditions, the three proposed baseline detectors are prone to incorrectly detect human posture in thermal images.

### 5.3. Ablation Study

**Figure 9:**
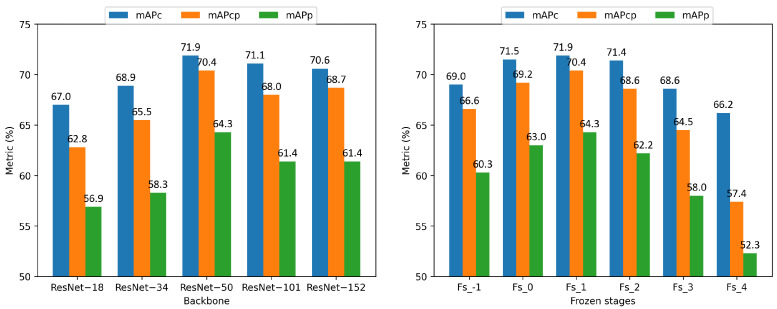
Illustration via bar chart of the effect of the backbone network (**left**) and frozen_stages (**right**) on the mAP metric on the IPHPDT dataset.

**Impact of the backbone network.** In order to study the influence of the backbone network for predicting human posture, we evaluate IPH-TOOD with different depths of backbone network on IPHPDT; moreover, we also evaluate IPH-TOOD with different frozen_stages in ResNet with a depth of 50 on IPHPDT. Specifically, the backbone network is a ResNet, whose depth values can be chosen from 18, 34, 50, 101, and 152, where ResNet-18 denotes the ResNet with a depth of 18, and whose frozen_stage values can be chosen from −1, 0, 1, 2, 3 and 4, where fs_−1 denotes the frozen_stage with a value of −1. Frozen_stages indicates that the network stage is frozen during network fine-tuning (i.e., the back-propagation algorithm is not performed during training), and the backbone in this experiment contains one stem and four stages. When frozen_stages is −1, the network is not frozen; when it is 0, the stem is frozen; when it is 1, the stem and first stage are frozen; when it is 4, the whole backbone is frozen. Table 5 and Table 6 display the mAPs and the APs at fixed IoUs (i.e., 0.5 and 0.75) of IPH-TOOD on IPHPDT with respect to different backbone networks and different frozen_stages based on ResNet-50, respectively. To aid with more intuitive understanding, the results of the mAP metric are plotted in the bar chart shown in Figure 9. As can be observed in Table 5, the AP is optimal when the depth equals 50, which is also the default setting in our paper, and when the depth of the backbone network goes from 18 to 50, all the APs and mAPs increase, but they decrease with fluctuations when the depth ascends from 50 to 152. That may be explained by the fact that increasing the depth of the backbone network can improve the representation power of the detector, but more training data are required to optimize the parameters of the backbone network as it becomes larger. As can be seen in Table 5, the AP is optimal when the frozen_stages equals 1, which is also the default setting in our paper, and when the frozen_stages of the backbone network goes from −1 to 1, all the APs and mAPs increase, but they decrease with fluctuations when the depth ascends from 1 to 4. That may be explained by the fact that the features of the first few layers are basic general features, they can save memory and accelerate convergence without re-training, but the last few layers have deeper features and need to be re-trained to learn more information. Experimental results suggest that ResNet of the depth of 50 and frozen_stages of the value of 1 are the optimal choice for finetuning the proposed detector IPH-TOOD for the proposed task, given the scale of the proposed dataset.

**Figure 10:**
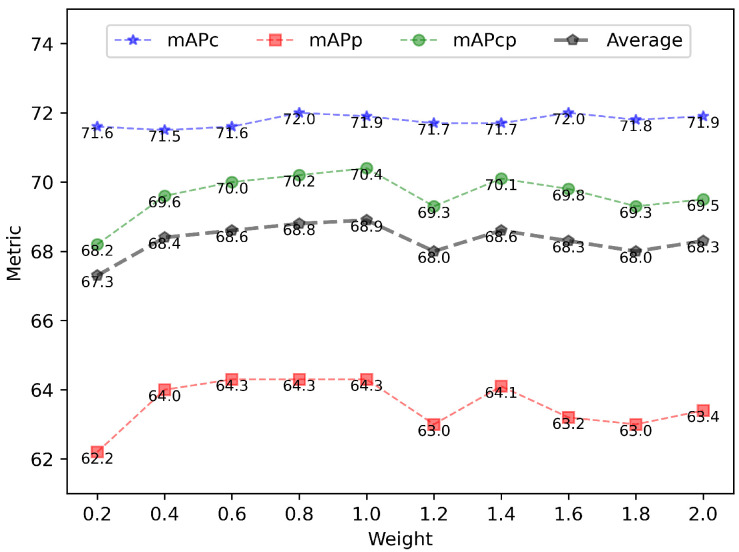
Illustration of trends in the values and means of the three indicators relative to the weighting coefficient for the loss of predicting human posture on the IPHPDT dataset.

**Weighting the loss of predicting human posture.** In order to understand the effect of the weighting coefficient for the loss of predicting human posture, we assess IPH-TOOD on IPHPDT with regard to the weighting coefficient, i.e., λ in Equation (7), which varies from 0.2 to 2.0 in steps of 0.2. Table 7 shows the mAPs and the APs at fixed IoUs (i.e., 0.5 and 0.75) of IPH-TOOD on IPHPDT. The trend of the average of the three metrics as the weight changes from 0.2 to 2.0 is plotted in Figure 10 to provide a more intuitive grasp of the influence of this weight. Note that the average of the three metrics is plotted by the gray dotted line. It can be observed that the best AP emerges between 0.6 and 1.6, yet all the best APs cannot be obtained concurrently at a fixed λ in IPH-TOOD. Overall, the change of λ has basically little effect on $AP_{c}$ as the difference between the maximum and minimum of $AP_{c}$ is not more than 0.5%. However, obvious variations can be observed on both $AP_{p}$ and $AP_{cp}$ as λ varies, and the changes of $AP_{p}$ and $AP_{cp}$ are basically synchronized, indicating that $AP_{c}$ is closely associated with $AP_{cp}$. We can also observe that the optimal $AP_{p}$ and $AP_{cp}$ occur when λ ranges from 0.6 to 1.0, and overall the maximum values are at λ = 1.0, which is also the default setting. In summary, we can conclude that $AP_{c}$s all reach the maximum when λ = 1.6, whereas $AP_{p}$s and $AP_{cp}$s basically reach their optimal value at λ = 1.0. This suggests that localizing humans and identifying human postures counteract when these two tasks are combined as a composite task. Better methods that are able to diminish this counteracting are desirable, which will be an important consideration in our future work.

## 6. Conclusions

In this paper, we formulate a new task for identity-preserved human posture detection in infrared thermal images, which is a compromise between human pose estimation and human detection for a threefold purpose. The first is to establish an identity-preserved task with thermal images; the second is to achieve more information than the location of persons as provided by human detection for more advanced computer vision applications; the third is to avoid difficulties collecting well-annotated data for human pose estimation in thermal images. This task underpins various applications where privacy matters and may also draw attention to more informative object detection other than identification and localization. We present the IPHDT dataset for infrared human posture detection and establish three baseline detectors based on state-of-the-art object detection models, i.e., IPH-YOLOF, IPH-YOLOX, and IPH-TOOD, to promote further exploratory research on this problem.

We believe that our work will attract greater attention to the study of human posture in the field of infrared thermal images, which is important for advanced application scenarios such as human detection based on privacy protection, elderly guardianship system, hospital care, etc. Nevertheless, there are some limitations of our work worthy of note. Although the posture types considered here are the most common ones, covering more posture types will be desirable in applications where more specific posture information is needed. In addition, the network architectures proposed for predicting human posture is relatively simpler, and more effective structures should be explored for better performance. In our future work, we will consider more types of human postures and explore better methods to diminish the counteracting between the tasks of localizing humans and identifying human postures and develop better detectors for detecting human posture in thermal images based on more state-of-the-art object detectors.

## Figures and Tables

**Table 1:** Comparison of advantages and disadvantages of our methods (i.e., IPH-YOLOF, IPH-YOLOX, and IPH-TOOD) with previous methods.

Method	Year	Dataset	Learning Method	Supervisio Method	YOLO	Attention	FPN	Posture Prediction Head
[40]	2013	OSU-T	Traditional learning	Supervised	×	*√*	×	×
[50]	2016	Non-public			×	×	×	×
[39]	2019	OSU IMS DIP			×	×	×	×
[51]	2018	Non-public	Traditional learning	Semi- supervised	×	*√*	×	×
[52]	2021	IRPSRL MS COCO	Deep learning		×	×	×	×
[53]	2018	Non-public	Deep learning	Unsupervised	×	×	×	×
[45]	2020	Non-public			*√*	×	*√*	×
[41]	2017	OSU-T OSU-CT LSI KAIST	Deep learning	Supervised	×	×	×	×
[47]	2021	OSU-T			*√*	*√*	*√*	×
[54]	2021	MPII-HPD AI-CD			×	*√*	×	×
IPH-YOLOF	2022	IPHPTD			*√*	*√*	×	*√*
IPH-YOLOX	2022	IPHPTD			*√*	*√*	*√*	*√*
IPH-TOOD	2022	IPHPTD			×	*√*	*√*	*√*

**Table 2:** Comparison between the IPHD dataset and our proposed IPHPDT dataset in terms of the number of images.

Dataset	Train Set	Valid Set	Test Set
IPHD	84,818	12,974	15,115
IPHPDT	62,010	-	13,267

**Table 3:** Illustration of the $AP_{p}$ difference between the three baseline detectors we proposed, i.e., IPH-YOLOF, IPH-YOLOX, and IPH-TOOD, on the IPHPDT dataset. Note that $AP_{c}$, $AP_{p}$, and $AP_{cp}$ represent the precision metric for the prediction of the human category, human posture, and the composite of both, respectively.

	{$AP_{c}$,$AP_{p}$,$AP_{cp}$}@0.5	{$AP_{c}$,$AP_{p}$,$AP_{cp}$}@0.75	{$mAP_{c}$,$mAP_{p}$,$mAP_{cp}$}
IPH-YOLOF	(0.944,**0.833**,**0.867**)	(0.848,0.768,0.834)	(0.706,0.630,0.692)
IPH-YOLOX	(**0.955**,0.804,0.836)	(**0.863**,0.737,0.771)	(**0.737**,0.625,0.677)
IPH-TOOD	(0.935,0.826,0.863)	(0.850,**0.771**,**0.836**)	(0.719,**0.643**,**0.704**)

**Table 4:** Illustration of the $mAP_{cp}$ difference between the three baseline detectors we proposed, i.e., IPH-YOLOF, IPH-YOLOX, and IPH-TOOD, on the IPHPDT dataset. Note that $mAP_{cp}$ represents the mean average precision for predicting the composite of the human category and its posture.

	Standing	Sitting	Lying	Bending
$mAP_{cp}$(IPH-YOLOF)	0.723	**0.666**	0.720	0.665
$mAP_{cp}$(IPH-YOLOX)	**0.743**	0.625	0.721	0.619
$mAP_{cp}$(IPH-TOOD)	0.737	0.652	**0.725**	**0.695**

**Table 5:** Illustration of how the AP metrics of IPH-TOOD change with regard to the depth of the backbone network on the IPHPDT dataset.

Backbone	{$AP_{c}$,$AP_{p}$,$AP_{cp}$}@0.5	{$AP_{c}$,$AP_{p}$,$AP_{cp}$}@0.75	{$mAP_{c}$,$mAP_{p}$,$mAP_{cp}$}
ResNet-18	(0.912,0.771,0.804)	(0.797,0.678,0.744)	(0.670,0.569,0.628)
ResNet-34	(0.924,0.776,0.831)	(0.820,0.694,0.777)	(0.689,0.583,0.655)
ResNet-50	**(0.935,0.826,0.863)**	**(0.850,0.771,0.836)**	**(0.719,0.643,0.704)**
ResNet-101	(0.925,0.792,0.835)	(0.839,0.728,0.798)	(0.711,0.614,0.680)
ResNet-152	(0.933,0.796,0.840)	(0.833,0.726,0.797)	(0.706,0.614,0.687)

**Table 6:** Illustration of how the AP metrics of IPH-TOOD change with regard to the frozen_stages of the backbone network on the IPHPDT dataset.

Frozen Stages	{$AP_{c}$,$AP_{p}$,$AP_{cp}$}@0.5	{$AP_{c}$,$AP_{p}$,$AP_{cp}$}@0.75	{$mAP_{c}$,$mAP_{p}$,$mAP_{cp}$}
fs_−1	(0.924,0.791,0.835)	(0.824,0.723,0.794)	(0.690,0.603,0.666)
fs_0	(0.932,0.811,0.851)	(0.847,0.751,0.817)	(0.715,0.630,0.692)
fs_1	**(0.935,0.826,0.863)**	**(0.850,0.771,0.836)**	**(0.719,0.643,0.704)**
fs_2	(0.934,0.804,0.847)	(0.847,0.744,0.814)	(0.714,0.622,0.686)
fs_3	(0.923,0.773,0.818)	(0.820,0.695,0.769)	(0.686,0.580,0.645)
fs_4	(0.902,0.718,0.740)	(0.785,0.622,0.678)	(0.662,0.523,0.574)

**Table 7:** Illustration of how the AP metrics of IPH-TOOD change with regard to the weighting coefficient for the loss of predicting human posture on the IPHPDT dataset.

λ	{$AP_{c}$,$AP_{p}$,$AP_{cp}$}@0.5	{$AP_{c}$,$AP_{p}$,$AP_{cp}$}@0.75	{$mAP_{c}$,$mAP_{p}$,$mAP_{cp}$}
0.2	(0.934,0.805,0.843)	(0.848,0.751,0.815)	(0.716,0.622,0.682)
0.4	(0.935,0.827,0.860)	(0.846,0.764,0.825)	(0.715,0.640,0.696)
0.6	(0.935,**0.828**,0.862)	(0.848,0.766,0.829)	(0.716,**0.643**,0.700)
0.8	(0.935,0.822,0.857)	(**0.850**,0.768,0.833)	(**0.720**,**0.643**,0.702)
1.0	(0.935,0.826,**0.863**)	(**0.850**,**0.771**,**0.836**)	(0.719,**0.643**,**0.704**)
1.2	(0.936,0.811,0.853)	(0.848,0.753,0.822)	(0.717,0.630,0.693)
1.4	(0.935,0.820,0.857)	(**0.850**,0.769,0.833)	(0.717,0.641,0.701)
1.6	(**0.937**,0.805,0.852)	(**0.850**,0.756,0.830)	(**0.720**,0.632,0.698)
1.8	(0.935,0.815,0.855)	(0.849,0.754,0.824)	(0.718,0.630,0.693)
2.0	(0.935,0.814,0.853)	(0.849,0.759,0.825)	(0.719,0.634,0.695)

## Data Availability

The data that support the fingings of this study are available from the corresponding auther upon reasonable request.
